# Fiber type specific tibialis anterior muscle atrophy and oxidative capacity reduction is contemporaneous with death of larger lumbar motor neurons in old rats

**DOI:** 10.14814/phy2.71035

**Published:** 2026-07-26

**Authors:** Kathryn D. Rooker, Genesis A. Hernandez‐Vizcarrondo, Sang Won Cheung, Sanjana Mahadev Bhat, Alyssa D. Brown, Gary C. Sieck, Matthew J. Fogarty

**Affiliations:** ^1^ Department of Physiology and Biomedical Engineering Mayo Clinic Rochester Minnesota USA; ^2^ Department of Neurology Mayo Clinic Rochester Minnesota USA

**Keywords:** motor unit, oxygen consumption, sarcopenia, succinate dehydrogenase

## Abstract

Age‐associated muscle weakness and atrophy of limb muscles, termed sarcopenia, is a major factor in the morbidity of the elderly. It is becoming increasingly recognized that a substantial contribution to the sarcopenic phenotype arises from motor neuron (MN) death in old age and subsequent muscle denervation. In human and rodent models, mitochondrial dysfunction is a leading culprit in both muscle and MN deterioration with age. In other motor pools within the Fischer 344 (F344) aging rat model, we showed that sarcopenia is selective to type IIx/b muscle fibers. We also showed a loss of larger MNs and denervation of the IIx/b fibers, which together comprise more fatigable fast (type FF) motor units. This selective vulnerability of larger MNs and type IIx/b muscle fibers to sarcopenia is further reflected by reductions in oxidative capacity of IIx/b fibers, as assayed by determining the maximum velocity of the succinate dehydrogenase reaction (SDHmax). Here, we hypothesize similar changes will occur in F344 tibialis anterior (TA) muscle from young (6‐months) and old (24‐months) groups. We also predict lumbar MN death in young and old F344 rats. We found selective atrophy of type IIx/b fibers in the TA from old rats. In old age, SDHmax was reduced but only in IIx/b TA fibers. These muscle observations were concomitant with the death of larger lumbar MNs in old age. This study indicates the remarkable selectivity of type FF TA motor units to age‐associated perturbations and sarcopenia.

## 1 INTRODUCTION

Sarcopenia, the age‐related loss of muscle specific force (force normalized for muscle cross‐sectional area [CSA]) and fiber atrophy, occurs in striated muscles, contributing to impaired motor performance with age (Fogarty, 2026; Fogarty et al., 2026; Larsson et al., 2019). Established human and rodent evidence indicates that type IIx/b fibers are most vulnerable to sarcopenia (Fogarty & Sieck, 2019; Larsson et al., 2019; Sieck & Fogarty, 2025). These type IIx/b fibers are innervated by larger motor neurons (MNs) and together comprise fast fatigable (type FF) motor units that generate greater forces, such as that required for ballistic maneuvers such as jumping. By comparison, type I and IIa fibers are innervated by smaller MNs comprising slow and fast fatigue resistant motor units (type S and FR), respectively, which produce lower force, albeit with greater endurance (Burke et al., 1971; Fogarty & Sieck, 2019; Heckman & Enoka, 2012). In aging humans and rats, reductions in the oxidative capacity of larger MNs and type IIx/b muscle fibers (Fogarty et al., 2026; Fogarty, Marin Mathieu, et al., 2020; Proctor et al., 1995; Rygiel et al., 2014; St‐Jean‐Pelletier et al., 2017) are consistent with mitochondrial dysfunctions triggering motor unit type‐dependent sarcopenia and aging neuromotor decline (Fogarty et al., 2026).

In a variety of motor pools, age‐related loss of MNs results in muscle weakening and selective atrophy of type IIx/b fibers (Larsson et al., 2019; Sieck & Fogarty, 2025; Willadt et al., 2018), a scenario strikingly redolent of denervation (Geiger et al., 2001; Geiger et al., 2003; Sieck et al., 2007). In other motor pools, we and others have shown that, in old age, the contractile and metabolic effects on type IIx/b fibers (Fogarty et al., 2019; Fogarty et al., 2026; Fogarty & Sieck, 2019; Hernandez‐Vizcarrondo et al., 2026; Khurram et al., 2018; Larsson et al., 2019; Rowan et al., 2012) are selective to behaviors requiring activation of IIx/b fibers (Fogarty, 2025; Fogarty & Sieck, 2026; Khurram et al., 2018). In Fischer 344 (F344) rats, a major model of aging in rodents, it is unknown whether concomitant age‐related loss of large lumbar MNs and atrophy of type IIx/b muscle fibers occurs. To date, limited evidence in male rats suggests that there is a selective loss of larger lumbar MNs (Cheung & Fogarty, 2026; Ishihara et al., 1987; Jacob, 1998). We chose the TA muscle as it has a high proportion of vulnerable type IIx/b fibers in rats (Pullen, 1977), and is known to experience denervation in aging humans (McNeil et al., 2005). We chose F344 rats as they exhibit the same variability and cognitive decline as the general aging human populations (Alexander et al., 2012; Bizon et al., 2012), and, of importance to neuromotor studies, exhibit similar spinal MN losses with age as humans (Cheung & Fogarty, 2026; Tomlinson & Irving, 1977). In different rat strains, there is evidence for an age‐related decrease in TA force production and/or muscle fiber atrophy (Ishihara et al., 1987; Larsson & Edstrom, 1986; Pannerec et al., 2016). In Wistar rats, lumbar MN loss and selective atrophy of the gastrocnemius is evident (Rowan et al., 2012). Despite a relative lack of empirical data, there is a prevailing thought that, in old age, skeletal muscle fibers are lost (Wilkinson et al., 2018), although this remains controversial (Ballak et al., 2014; Koseki et al., 1998; Schon & Manfredi, 2003). Indeed, under conditions where the number of MNs surviving development is manipulated, muscle fiber populations remain constant (Brandenburg et al., 2024; Fogarty, Brandenburg, & Sieck, 2020; Fogarty, Sieck, & Brandenburg, 2020).

In previous studies, we validated a quantitative histochemical technique to measure the maximum velocity of the succinate dehydrogenase reaction (SDHmax) in muscle fibers (Sieck et al., 1986). SDH is located in the inner mitochondrial membrane and acts both as a key enzyme in the tricarboxylic acid (TCA) cycle and as complex II in the electron transport chain (Brown, Fogarty, & Sieck, 2021; Sieck et al., 1995; Sieck et al., 1996). SDH mediates the oxidation of succinate to fumarate in the TCA cycle, which is coupled to the reduction of ubiquinone to ubiquinol in the electron transfer chain. We previously measured SDHmax in muscle fibers under a variety of conditions to evaluate changes in mitochondrial oxidative capacity specific to muscle fiber type, rather than whole tissue respirometry‐based measurements of O_2_ consumption rate (Fogarty et al., 2026; Sieck et al., 1986; Sieck et al., 1995; Sieck et al., 1996). SDHmax is higher in type I and IIa fibers compared to type IIx/b fibers in multiple muscles (Blanco et al., 1988; Blanco et al., 1991). In old age, SDHmax of type IIx/b fibers is specifically reduced in tongue and diaphragm (Fogarty et al., 2026; Fogarty, Marin Mathieu, et al., 2020). In aging MNs, we also found a selective reduction in SDHmax in larger (likely type FF) MNs (Fogarty et al., 2026).

In the present study, we hypothesize that there is a selective age‐related atrophy and reduction of SDHmax in type IIx/b TA fibers. We further hypothesize that these muscle changes will be associated with a loss of larger lumbar MNs. In this study, we are the first to show fiber type specific IIx/b quantitative reductions of SDH in old age hindlimb muscle, consistent with contractile dysfunctions in these fibers (Larsson et al., 2019). Here, we use unbiased stereological methods that are essential for accurately quantifying age‐related MN loss, as they minimize biases associated with regional sampling (Slomianka, 2020). Despite the importance of rigorous MN quantification, only one study has previously combined stereological lumbar MN counts and anatomical hindlimb muscle analysis in rodents (Blasco et al., 2020; Cheung & Fogarty, 2026). This study further advances the field by integrating stereological lumbar MN survival with measures of TA muscle oxidative capacity, providing a comprehensive evaluation of both structural and physiological changes in the aging motor unit of females and males.

## 2 METHODS

### 2.1 Ethical approval

All animal use and protocols were aligned with best practice (Drummond, 2009) and approved by the Mayo Clinic Institutional Animal Care and Use Committee (IACUC #A00008010‐24) and complied with NIH guidelines, State and Federal laws.

### 2.2 Animals

Young (6‐months old; *n* = 13 [7 females, 6 males]) and old (24‐months old; *n* = 12 [6 females, 6 males]) female and male Fischer 344 (F344) pathogen‐free rats were obtained from the NIH aged rodent colony. These ages were selected based on survival information (100% and 50%, respectively), with modest delays in females compared to males (Turturro et al., 1999). Animals were maintained on an alternating 12:12 h light–dark cycle with ad libitum access to fresh water and rat chow (Rodent LaboratoryDiet Cat # 5001, Lab Diet). Rats were housed in pairs in an effort to control for socialability and cage activity. An acclimation period of at least 48 h was provided before conducting any experimental procedures, and animals were weighed weekly until the terminal experiment was performed.

### 2.3 Muscle preparations

**FIGURE 1:**
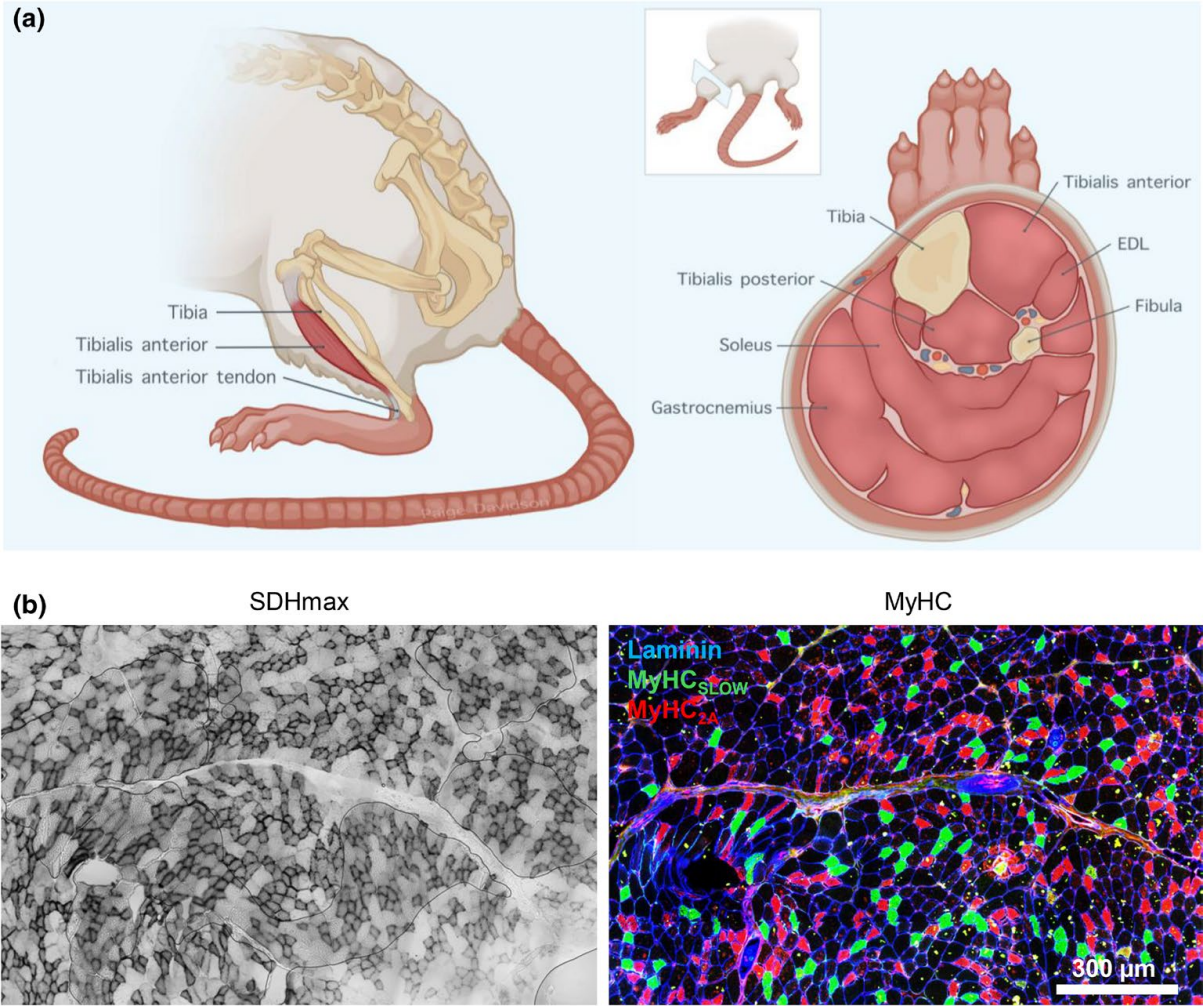
Experimental approach. (a) In F344 rats we evaluated the tibialis anterior muscle, a major dorsiflexor of the hind‐paw (left side image), with gross and fiber morphometry assessed at the mid‐belly region (right sided image). (b) Example serial sections with SDHmax gray level quantitative histochemistry (left image) and myosin heavy chain (MyHC) labelling (right image) of MyHC_slow_ (green, to identify type I fibers) and MyHC_2A_, (red, to identify type IIa fibers) along with fiber borders bounded by laminin (blue).

In a subset of rats (*n* = 7 young; *n* = 6 old), following deep ketamine (80 mg/kg) and xylazine (20 mg/kg) intraperitoneal anesthesia, rats were euthanized by exsanguination. The left TA was exposed and blunt dissected away from the surrounding muscles from origin to insertion (Figure 1a), excised, stretched to 150% of resting length to approximate optimal length (*L*_o_) for contractions (Fogarty, Sieck, & Brandenburg, 2020) and fresh‐frozen on cork in melting isopentane cooled by liquid nitrogen, in a manner identical to past reports (Fogarty, Sieck, & Brandenburg, 2020). These frozen muscle strips were stored at −80°C until processed further for either SDHmax measurements or fiber typing.

### 2.4 Measuring SDHmax

Transverse tibialis anterior serial sections were cut at 8 μm thickness using a cryostat (Reichert Jung Frigocut 2800 Cryostat, Reichert Microscope Services, Depew, NY, USA). The SDH reaction was performed on sections incubated for 8 min (end‐point of the reaction) in a solution containing 80 mM succinate, 1.5 mM nitro blue tetrazolium (NBT – reaction indicator), 5 mM EDTA, 0.2 mM mPMS, and 0.1 mM azide in 0.1 M phosphate buffer (pH = 7.6) (Sieck et al., 1986). Images were acquired using a Nikon A1R confocal microscope (RRID:SCR_020317; Nikon Instruments Inc., Melville, NY) with a 60×/1.4 NA oil‐immersion objective at 12‐bit resolution into a 1024 × 1024‐pixel array. An interference filter with a peak emission wavelength of 570 nm was placed in the transmitted light path to limit the spectral range of the light source to the maximum absorption wavelength of nitro blue tetrazolium diformazan (NBT_dfz_). The measured gray levels (GL) of the microscope were calibrated to known optical density (OD) units using a photographic density step‐wedge tablet (0.04–2.20 OD units in increments of 0.15 OD; Stouffer Industries, Mishawaka, IN). The dynamic range of the microscope was adjusted to take advantage of the full range of OD while avoiding saturation of the images at both ends of the OD range, as described previously (Sieck et al., 1986). Tibialis anterior fibers were matched to serial sections used to identify fiber type (see below) to stratify SDHmax based on classification as type I, type IIa, or type IIx/b fibers (Figure 1b). The change in OD within the selected muscle fiber region of interest was measured by comparing to a serial section of the same TA fiber incubated in a solution without succinate. From this, the SDHmax (in mmol fumarate L fiber^−1^ min^−1^) was then determined using the Beer–Lambert equation (below)

$$\text{SDHmax}=\frac{d\left[{\mathrm{NBT}}_{\mathrm{dfz}}\right]}{\mathrm{dt}}=\frac{\frac{\mathrm{dOD}}{\mathrm{dt}}}{\mathrm{kl}}$$

where the accumulation of reaction product (d[NBT_*dfz*_]/dt) is determined by the change in OD (dOD/dt) across a pathlength (muscle fiber cross‐sectional thickness – 8 mm) times the molar extinction coefficient for NBT_dfz_ (26,478 mol^−1^ L cm^−1^).

### 2.5 Tibialis anterior muscle fiber types

In serial sections (of the same fibers) adjacent to those used for determining SDHmax, fiber type was determined based on immunoreactivity to different myosin heavy chain (MyHC) isoforms as previously described (Brown, Davis, et al., 2021; Fogarty, Marin Mathieu, et al., 2020; Sieck et al., 1996) (Figure 1b). Briefly, sections were fixed in acetone for 10 min prior to commencing immunofluorescence staining protocols. Sections were blocked for 30 min in 10% goat serum and incubated overnight at 4°C in primary antibodies for the following MyHC isoforms: MyHC_Slow_ (BA‐F8, 1:3 dilution; Developmental Studies Hybridoma Bank, Iowa City, IA), MyHC_2A_ (SC‐71, 1:3 dilution; Developmental Studies Hybridoma Bank). Fluorescently conjugated secondary antibodies were then applied at a 1:200 dilution, using Alexa Fluor 488 (Cat # A‐11001, Thermo Fischer) to visualize MyHC_Slow_ and Alexa Fluor 568 (Cat # A‐21043, Thermo Fischer) to visualize MyHC_2A_. Based on the staining pattern, TA fibers were classified as type I, type IIa, and type IIx/b. Immunolabeled TA cross‐sections were imaged using a 20× oil‐immersion objective (NA 1.0) on an Olympus FV2000 (Olympus USA) laser confocal microscope with a 1024 × 1024 pixel array, with similar acquisition parameters across preparations. A mosaic of the entire TA was imaged for gross morphometric assessments. The morphometric parameters of the TA muscle were determined using ImageJ tools, in a manner identical to past reports (Fogarty, Marin Mathieu, et al., 2020; Fogarty & Sieck, 2021).

### 2.6 Lumbar MN counting

Following euthanasia, a cohort of rats (*n* = 5 per age group) was transcardially perfused with heparinized saline followed by 4% paraformaldehyde (PFA) in 0.1 M phosphate‐buffered saline (PBS, pH 7.4). The fixed lumbar spinal cord was excised, post‐fixed in 4% PFA overnight, and then immersed overnight in 25% sucrose in PBS.

Serial transverse (16 μm) cryosections of the lumbar spinal cord were cut and stained with 0.1% thionin (v/v) in an acetic acid buffer using previously established methods (Fogarty et al., 2013; Fogarty et al., 2015). Using a Zeiss Axoskop II with a 20× air objective (1.0 NA, Zeiss, Gottingen, Germany), lumbar MN numbers were quantified unilaterally in the left lumbar motor enlargement (every 10th section) (Fogarty et al., 2013; Fogarty et al., 2015; Steyn et al., 2013), between L1 and L6 (Sengul et al., 2012). To qualify for counting, MNs were identified as large cells (the longest diameter >30 μm) (Ferrucci et al., 2018), having a dark cytoplasm, a distinct pale nucleus and dark nucleoli, as outlined previously (Ferrucci et al., 2018). Fragments of MNs, without a nucleus and nuclei, were not counted to avoid “double counting”, consistent with stereological principles (Ferrucci et al., 2018; Slomianka, 2020).

### 2.7 Statistical methods

All statistical analyses were performed using PRISM software (GraphPad PRISM Version 10.2.3, La Jolla, CA). Statistical significance was established at the *p* < 0.05 level, reported precisely to three decimal places. All data are presented as mean ± the standard deviation (SD) and assessed for normality with Shapiro–Wilk tests. For all comparisons, unpaired *t*‐test, Mann–Whitney *U*‐Tests, or 2‐way ANOVAs with Bonferroni post hoc tests, respectively, were used when warranted. Kolmogorov–Smirnov tests were used to compare frequency distributions between populations. To ensure statistical differences were meaningful, reliable, and robust, we reported Cohen's *d* (Gandevia, 2021). We had a priori exclusion criteria for replicate data, namely, all data greater than 2.5 standard deviations from the mean were removed. Experiments, imaging, and analyses were performed blind to age and sex. We did not power to detect sex differences in aging and included female and male rats to mirror the heterogeneity of the aging human population. Major outcome measures stratified for sex are presented in tabular form (Table 1).

**TABLE 1:** Sex‐stratified outcome measures.

Parameter	Young (*n*)	Old (*n*)	% difference	*p* Value
Total TA CSA (mm^2^)	♀: 8.66 ± 1.19 (4) ♂: 10.6 ± 1.56 (3)	♀: 6.78 ± 1.59 (3) ♂: 8.74 ± 0.17 (3)	Young: 10.8% Old: 8.0%	Sex: *p = 0.022 Age: *p = 0.026
TA Fiber #	♀: 2566 ± 217 (4) ♂: 2288 ± 70 (3)	♀: 2507 ± 429 (3) ♂: 2706 ± 170 (3)	Young: 22.3% Old: 28.8%	Sex: *p* = 0.788 Age: *p* = 0.236
TA Fibers per mm^2^	♀: 300 ± 41 (4) ♂: 220 ± 40 (3)	♀: 346 ± 39 (3) ♂: 310 ± 20 (3)	Young: 26.6% Old: 10.6%	Sex: *p = 0.020 Age: *p = 0.009
Type I Fiber CSA (μm^2^)	♀: 1167 ± 86 (4) ♂: 1430 ± 86 (3)	♀: 1153 ± 182 (3) ♂: 1144 ± 226 (3)	Young: 22.6% Old: 1.7%	Sex: *p* = 0.167 Age: *p* = 0.109
Type IIa Fiber CSA (μm^2^)	♀: 1340 ± 158 (4) ♂: 1617 ± 435 (3)	♀: 1268 ± 468 (3) ♂: 1404 ± 15 (3)	Young: 20.7% Old: 1.0%	Sex: *p* = 0.271 Age: *p* = 0.440
Type IIx/b Fiber CSA (μm^2^)	♀: 3069 ± 318 (4) ♂: 4008 ± 620 (3)	♀: 2143 ± 540 (3) ♂: 2586 ± 140 (3)	Young: 30.6% Old: 20.7%	Sex: *p = 0.019 Age: *p = 0.001
Type I Fiber SDHmax	♀: 2.97 ± 0.17 (4) ♂: 3.23 ± 0.78 (3)	♀: 3.09 ± 0.43 (3) ♂: 3.08 ± 0.78 (3)	Young: 8.6% Old: 0.3%	Sex: *p* = 0.707 Age: *p* = 0.959
Type IIa Fiber SDHmax	♀: 2.99 ± 0.53 (4) ♂: 2.41 ± 0.04 (3)	♀: 2.29 ± 0.47 (3) ♂: 2.32 ± 0.30 (3)	Young: 19.5% Old: 1.6%	Sex: *p* = 0.256 Age: *p* = 0.122
Type IIx/b Fiber SDHmax	♀: 1.51 ± 0.46 (4) ♂: 1.47 ± 0.57 (3)	♀: 0.63 ± 0.34 (3) ♂: 0.57 ± 0.39 (3)	Young: 2.3% Old: 9.2%	Sex: *p* = 0.859 Age: *p = 0.006
Lumbar MN #	♀: 4993 ± 571 (3) ♂: 4873 ± 205 (3)	♀: 3727 ± 855 (3) ♂: 3567 ± 669 (3)	Young: 2.4% Old: 4.3%	Sex: *p* = 0.717 Age: *p = 0.007
Lumbar MN Soma SA (μm^2^)	♀: 4553 ± 401 (3) ♂: 4760 ± 953 (3)	♀: 5007 ± 544 (3) ♂: 5012 ± 1260 (3)	Young: 4.6% Old: 0.1%	Sex: *p* = 0.836 Age: *p* = 0.497

*Note*: All analyses 2‐Way ANOVA. All data mean ± SD.

## 3 RESULTS

### 3.1 Gross morphological characteristics of aged tibialis anterior muscles

**FIGURE 2:**
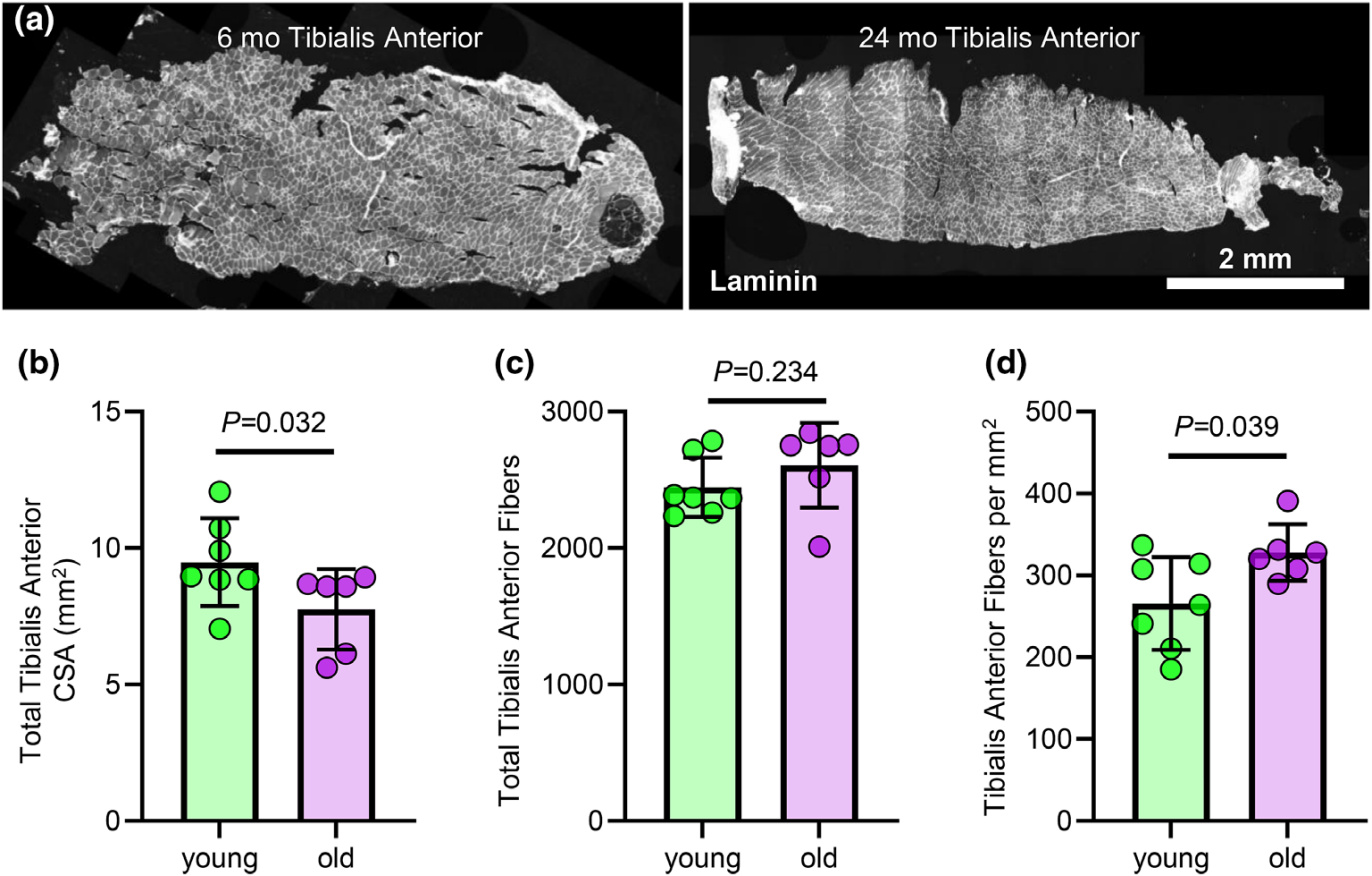
Gross morphometry of tibialis anterior muscles. (a) Tibialis anterior gross anatomy was evaluated in young (*n* = 7) and old (*n* = 6) ex vivo F344 rats, stretched to ~Lo. (b) Scatterplot (mean ± SD) of reduced overall tibialis anterior CSA (mm^2^) in old compared to young rats (Mann–Whitney *U*‐test). (c) Scatterplot (mean ± SD) showing unchanged total number of tibialis anterior muscle fibers in young and old rats (Student's unpaired *t*‐test). (d) Scatterplot (mean ± SD) showing increased tibialis anterior muscles fiber density (fibers per mm^2^) in old compared to young rats (Student's unpaired *t*‐test). Each dot represents an individual rat (i.e., the *n*).

General morphological characteristics of the entire TA muscle were evaluated in young and old rats at the mid‐belly portion of the muscle in a subset of F344 rats (*n* = 7 young; *n* = 6 old; Figure 2a). Overall muscle CSA of the TA muscle was reduced by 18% (*p* = 0.032; Cohen's *d* = 1.12, a large effect) in old (9.49 ± 1.61 mm^2^) compared to young (7.76 ± 1.47 mm^2^) F344 rats (Mann–Whitney *U*‐test; Figure 2b). The total number of TA muscle fibers did not differ between young (2447 ± 217) and old (2607 ± 311) F344 rats (*p* = 0.234; Student's unpaired *t*‐test; Figure 2c). In older TA muscles, total muscle fiber density increased by 24% (*p* = 0.039; Cohen's *d* = 1.30, a large effect) in old (328.1 ± 34.4 per mm^2^) compared to young (265.7 ± 56.7 per mm^2^) F344 rats (Student's unpaired *t*‐test; Figure 2d).

When sex was taken into account, gross TA CSA was larger in males compared to females, with proportional decreases ~25% in both sexes with age (Table 1). No sex differences were observed in the total TA fiber number (Table 1). In general, females had a greater fiber density per TA CSA, with increases in fiber density more exaggerated in males compared to females (Table 1), presumably due to greater relative atrophy of IIx/b fibers (see below).

### 3.2 Selective atrophy of type IIx/b fibers in aged tibialis anterior muscles

**FIGURE 3:**
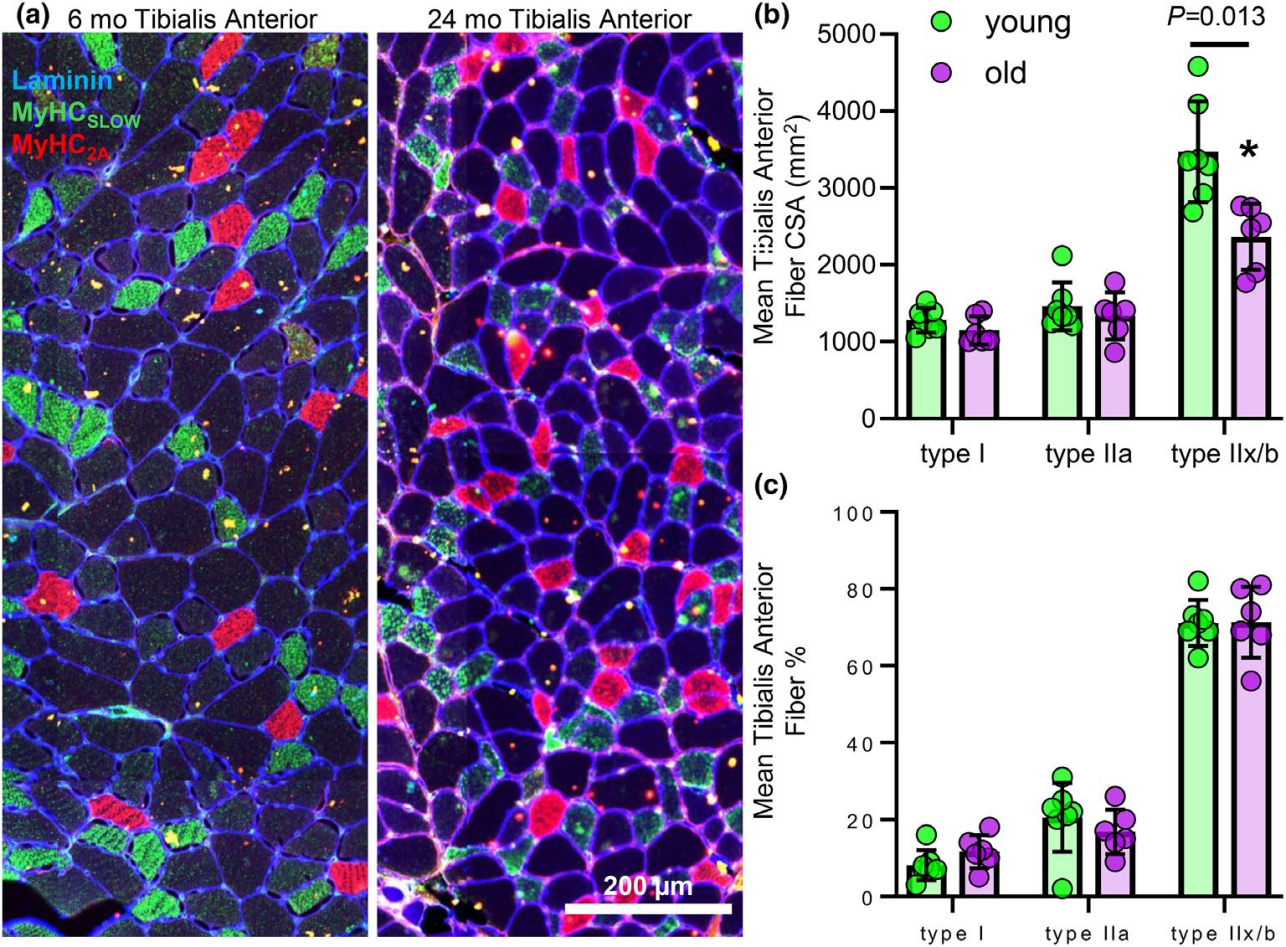
Fiber type specific atrophy of IIx/b fibers in old tibilais anterior muscle. (a) Type I (identified by MyHC_slow_—green), IIa (identifed by MyHC_2A_—red), and IIx/b fiber CSAs were evaluated in young (*n* = 7) and old (*n* = 6) ex vivo tibialis anterior muscle, stretched to Lo. Lamini (blue) was used to circumscribe each fiber border. (b) Scatterplot (mean ± SD) of reduced type IIx/b fiber CSA (Bonferroni post‐test) in old age, with unchanged type I and IIa CSA (2‐way ANOVA; Age: *p* = 0.015; Fiber Type: *p* < 0.0001; Interaction: *p* = 0.001). (c) Scatterplot (mean ± SD) showing unchanged fiber type % in young and old rats (2‐way ANOVA; Age: *p* = 0.915; Fiber Type: *p* < 0.0001; Interaction: *p* = 0.539). Each dot represents an individual rat (i.e., the *n*).

Individual fiber CSAs were evaluated in young (*n* = 7) and old (*n* = 6) F344 rat TA muscles (Figure 3a). Major determinants of muscle CSA were age (*F* = 8.4; *p* = 0.015), fiber type (F = 110.0; *p* < 0.0001) and the interaction between age and fiber type (*F* = 10.1; *p* = 0.001; 2‐way ANOVA; Figure 3b). Bonferroni post tests revealed there were no significant age‐related changes in type I (*p* = 0.608) or type IIa fiber (*p* > 0.99; Figure 3b) CSAs. By contrast, the CSA of type IIx/b TA muscle fibers was reduced by 32% (*p* = 0.013; Cohen's d = 1.96, a large effect) in old (2365 ± 428 μm^2^) compared to young (3472 ± 656 μm^2^) F344 rats (Figure 3b).

When sex was taken into account, no sex nor age differences were observed in type I nor type IIa fiber CSA (Table 1). However, in type IIx/b fibers, males had larger CSAs compared to females within both age groups, with age‐related reductions disproportionate in male F344 rats (Table 1). It should be noted that old male type IIx/b TA CSAs were smaller than those of young females (Table 1).

Major determinants of TA fiber type % were fiber type (*F* = 211.8; *p* < 0.0001) but not age (*F* = 0.9; *p* = 0.915) nor age and fiber type interactions (*F* = 0.6; *p* = 0.539; 2‐way ANOVA; Figure 3c). Bonferroni post tests revealed that in both young and old rats, the % of type IIx/b fibers was 4‐ to 5‐fold greater than type I and type IIa fibers (Figure 3c).

### 3.3 Reduced SDHmax in type IIx/b tibialis anterior fibers

**FIGURE 4:**
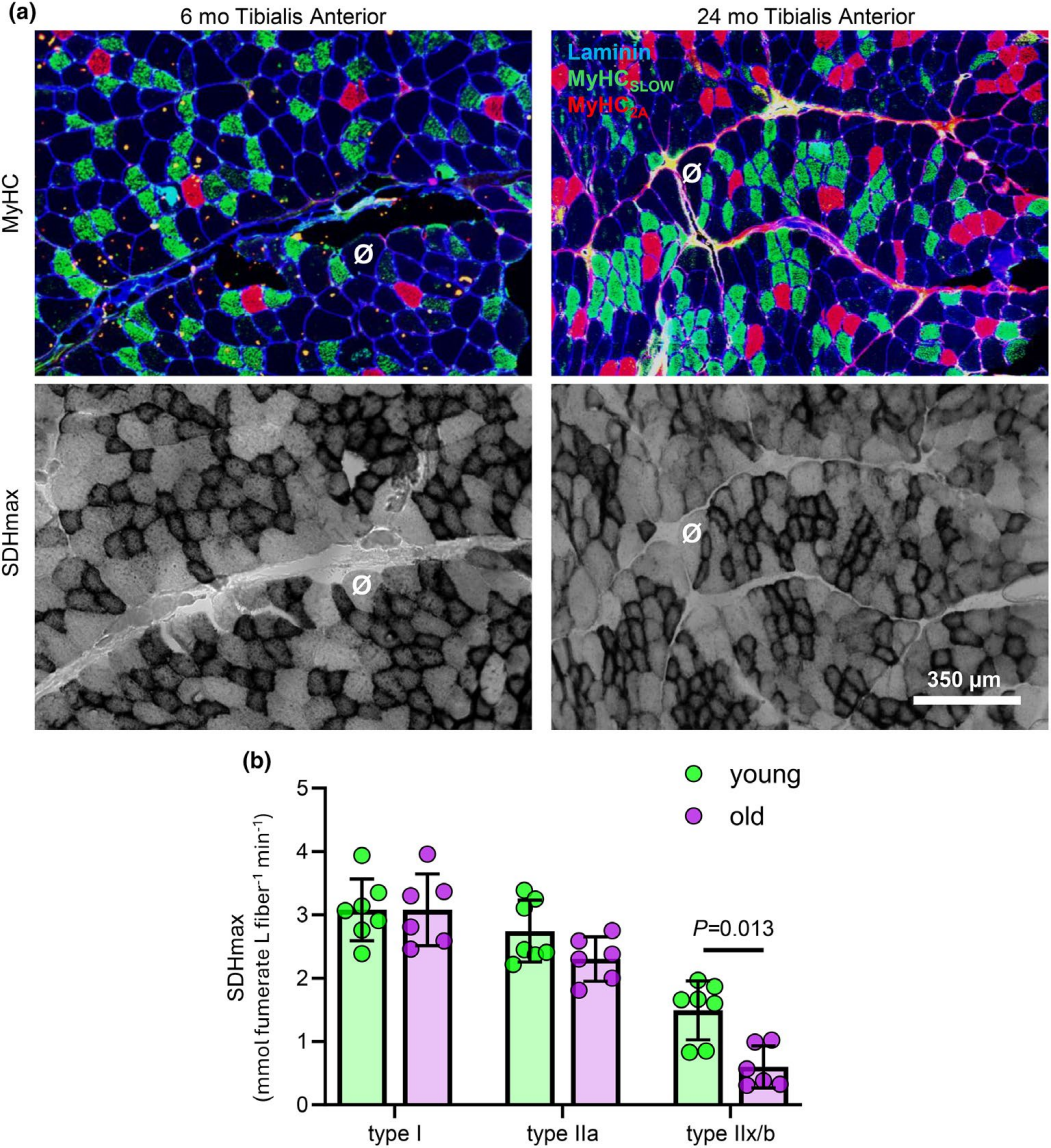
Fiber type specific reduction in SDHmax of IIx/b fibers in old tibilais anterior muscle. (a) Pictomicrographs show SDH activity after 8 min and fiber type classification in serial sections from young (*n* = 7) old (*n* = 6) rats. Identification of individual fibers is aided by anchoring to single readily verifiable fibers (Ø). (b) Scatterplot showing SDHmax (mmol fumerate L fiber^−1^ min^−1^, mean ± SD) is reduced in type IIx/b fibers (Bonferroni post‐test) in old age, with unchanged SDHmax in type I and IIa fibers (2‐way ANOVA; Age: *P* = 0.027; Fiber Type: *p* < 0.0001; Interaction: *p* = 0.037). Each dot represents an individual rat (i.e., the *n*).

The SDHmax was quantified in type I, IIa and IIx/b TA muscle fibers of young (*n* = 7) and old (*n* = 6) F344 rats (Figure 4a). Major determinants of TA SDHmax were age (*F* = 6.5; *p* = 0.027), fiber type (*F* = 85.3; *p* < 0.0001) and the interaction between age and fiber type (*F* = 3.9; *p* = 0.037; 2‐way ANOVA; Figure 4b). Bonferroni post tests revealed there were no significant age‐related changes in the SDHmax of type I (*p* > 0.99) or type IIa TA fibers (*p* = 0.265; Figure 4b). By contrast, the SDHmax of type IIx/b TA fibers was reduced by 45% (*p* = 0.013; Cohen's *d* = 2.40, a large effect) in old (0.60 ± 0.30 mmol fumerate L fiber^−1^ min^−1^) compared to young (1.49 ± 0.43 mmol fumerate L fiber^−1^ min^−1^) F344 rats (Figure 4b).

When sex was taken into account, no sex nor age differences were observed in type I nor type IIa fiber SDHmax (Table 1). In type IIx/b fibers, age‐related reductions in SDHmax occurred in the absence of a sex effect (Table 1).

### 3.4 Reduced lumbar MN survival in old rats

**FIGURE 5:**
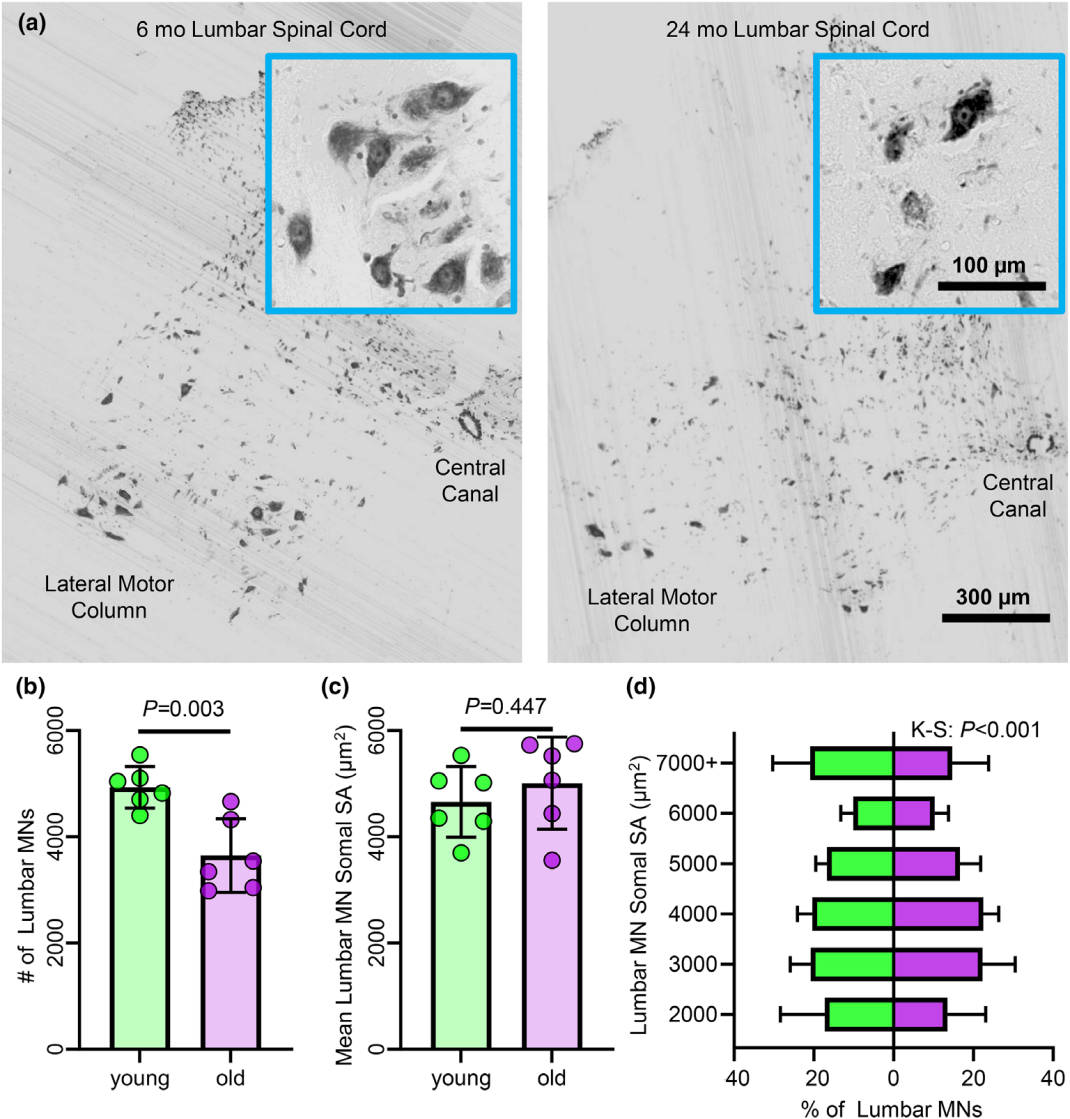
Loss of larger lumbar MNs in old rats. (a) Low and high powered pictomicrographs of the lumbar lateral motor column from young (*n* = 6) and old (*n* = 6) rats. (b) Scatterplot (mean ± SD) of reduced lumbar MN survival in old compared to young rats (Student's unpaired *t*‐test). (c) Scatterplot (mean ± SD) of unchanged lumbar MN mean surface area in young and old rats (Student's unpaired *t*‐test). (d) Frequency distribution plot showing disproportionate reduction in larger lumbar MNs in old rats (*p* < 0.001; Kolmogorov–Smirnov test). Each dot represents an individual rat (i.e., the *n*).

Lumbar MN survival was evaluated in young (*n* = 6) and old (*n* = 6) F344 rats using nissl stained transverse spinal cord sections (Figure 5a). In old rats, lumbar MN number (3647 ± 693) was reduced by 26% (*p* = 0.003; Cohen's *d* = 2.29, a large effect) compared to young (4933 ± 390) control rats (Student's unpaired *t*‐test; Figure 5b). We did not observe a sex effect in the age‐related reduction of total lumbar MNs (Table 1).

In old rats, the mean somal surface area of lumbar MNs was unchanged between young (4656 ± 664 μm^2^) and old (5009 ± 868 μm^2^) F344 rats (*p* = 0.447; Student's unpaired *t*‐test; Figure 5c). When looking at the overall frequency distribution histogram, a Kolmogorov–Smirnov test revealed a disproportionate reduction in larger lumbar MNs in old F344 rats (*p* < 0.001; Figure 5d). Mean lumbar MN somal SA was not affected by sex or age in our study (Table 1).

## 4 DISCUSSION

There are five major findings from the present study: (i) the gross CSA of the TA muscle decreases in old age, without the loss of individual muscle fibers; (ii) age‐related atrophy of individual TA fibers was confined to type IIx/b fibers; (iii) age‐related reduction in SDHmax was confined to type IIx/b fibers; (iv) age‐related reduction in the number of lumbar MNs disproportionately affects larger MNs that innervate IIx/b fibers (fast, fatigable motor units, type FF); and (v) while a marked sexual dimorphism was apparent in our TA morphological outcome measures, TA SDH and lumbar MN parameters were not affected by sex. Taken together, our results support the preponderance of evidence that age‐associated atrophy of the TA muscle is predominantly due to specific reductions in the CSA of type II/b fibers and not due to reductions in the number of muscle fibers. The selective atrophy of type IIx/b fibers and the reduced oxidative capacity (SDHmax) of these fibers is consistent with a denervation effect and with prior findings in other aged F344 striated muscles (Fogarty et al., 2026; Fogarty & Sieck, 2019; Gosselin et al., 1994; Miyata et al., 1995; Rowan et al., 2012; Sieck et al., 2023).

### 4.1 The effects of aging on striated muscle fibers

Muscle atrophy is one of the major tenets of sarcopenia, and here we observed an ~20% reduction in the TA CSA, at the point where the muscle belly is broadest. This gross morphological finding is similar to observations of humans, where year on year incremental reductions in muscle dimensions are noted (Ballak et al., 2014; Larsson et al., 2019), and gross atrophy in the rats' hindlimb (Ballak et al., 2014; Ishihara et al., 1987; Larsson & Edstrom, 1986; Pannerec et al., 2016; Rowan et al., 2012). This finding occurred in the absence of any frank loss of TA muscle fibers, with total fiber number remaining unchanged (Ballak et al., 2014; Koseki et al., 1998; Schon & Manfredi, 2003). Our findings of sexual dimorphism in TA morphology are consistent with reports of sex differences in a variety of muscles in F344 rats (Schenk et al., 2024).

It has been apparent for some time that under different conditions such as aerobic and resistance training, and denervation and immobilization, muscle fiber types respond differently and have differing adaptability (Kletzien et al., 2020; Proctor et al., 1995; Sieck et al., 2025; Zhu et al., 2025). In aging, the preponderance of evidence suggests that type IIx/b muscle fibers are more vulnerable to atrophy, while type I and IIa fibers are relatively unscathed (Fogarty & Sieck, 2019; Larsson et al., 2019). Whether this atrophy confers deficits in specific force and motor behaviors related to the recruitment of type FF motor units, such as the “get up and go” test in humans, is currently under investigation. In other striated muscles, we have shown that selective atrophy of type IIx/b fibers in aging (Fogarty et al., 2026; Gosselin et al., 1994; Sieck et al., 2023) is related to reduced specific force (force normalized to muscle CSA) and deficits in behaviors that require the recruitment of type FF motor units (Fogarty, 2025; Khurram et al., 2018).

### 4.2 The fiber type specific effects of aging on SDHmax in skeletal muscle

Consistent with the selective effects on type FF motor units in humans and rodent models (Fogarty & Sieck, 2019; Larsson et al., 2019), we observed a significant decrease of SDHmax in aged type II/b TA fibers. This is highly consistent with selective reductions in SDHmax in other brainstem and spinally innervated IIx/b muscle fibers (Brown, Davis, et al., 2021; Fogarty et al., 2026; Fogarty, Marin Mathieu, et al., 2020) in F344 rats and with studies in vastus lateralis muscle in older humans (Proctor et al., 1995). Our finding closely resembles the effect of muscle denervation, where IIx/b fibers are more severely afflicted in terms of mitochondrial reduction (Sieck et al., 2007). Our results are in contrast to the mouse TA muscle, where SDHmax has been shown to increase with age in all fiber types, although this was contemporaneous with fiber‐type conversion (Fattoretti et al., 2001). Notably, due to a lack of lumbar MN death in aged mice (Chai et al., 2011), a type‐specific denervation effect on SDHmax may not be present (Fattoretti et al., 2001), unlike in rats (Ishihara et al., 1987; Jacob, 1998) or humans (Cruz‐Sanchez et al., 1998; Kawamura et al., 1977; Terao et al., 1996; Tomlinson & Irving, 1977), where lumbar MN death is readily observed. There is some debate as to whether SDHmax represents the maximum oxidative capacity of the TCA cycle, with exercised human vastus lateralis having oxoglutarate dehydrogenase as the rate‐limiting enzyme (Blomstrand et al., 1997). Of note, in fatigued compared to non‐fatigued scenarios, SDHmax is reduced, suggesting that SDHmax declines may be related to submaximal under high activity scenarios (Johnson & Sieck, 1993). In this study, we are not constrained by biological limits of succinate substrate (Brown, Fogarty, & Sieck, 2021), where SDHmax is highly reflective of oxidative capacity.

These deficits in SDHmax are consistent with mitochondrial dysfunctions being a prime pathophysiological driver in age‐related neuromotor dysfunction. Mitochondrial defects have been noted in many studies in skeletal muscle (Lanza & Nair, 2009; Larsson et al., 2019; Leduc‐Gaudet et al., 2015; Short et al., 2005), and MN mitochondrial alterations with age have been under increasing scrutiny (Christensen & Fogarty, 2026; Fogarty et al., 2026). Indeed, in serial diaphragm muscle sections where SDH and mitochondrial volume were assessed within the same fibers, SDH production normalized to the volume of mitochondria was reduced in old age only in IIx/b fibers (Brown, Davis, et al., 2021), a result that may not be detected using tissue‐level evaluations (e.g., oroborous and seahorse). This result indicated intrinsic defects in aging mitochondria with fibers vulnerable to sarcopenia. The fact that skeletal muscle fibers and MNs are post mitotic increases the burden that any defects in cellular organelles have, as the cell cannot enter mitosis and turn over. Thus, efforts to optimize mitophagy and preserve proteostasis in skeletal muscle (and MNs) may be of therapeutic benefit in sarcopenia. Indeed, there is some evidence to suggest that even in the control state, mitochondria within type IIx/b fibers are in a more fragmented state than type I and IIa fibers, making them more prone to degenerative fragmentation and imbalanced fusion/fission signaling. In brainstem and tongue muscles, we have shown that systemic escalations in inflammatory cytokines are sufficient to drive a neuronal and muscular unfolded protein response that has type‐specific outcomes in SDH in muscle, and size‐dependent outcomes in MNs, consistent with type FF vulnerability (Fogarty et al., 2026).

### 4.3 Lumbar MN loss in aging and impaired limb muscle innervation

The lumbar MNs are somatotopically organized into ventral horn motor columns, exhibiting somatotopy such that MNs in the ventromedial portion innervate axial and proximal lower/hindlimb muscles, and those of the ventrolateral column innervate the distal muscles of the lower/hindlimbs (Bacskai et al., 2014; McHanwell & Watson, 2009; Sengul et al., 2012; Watson et al., 2009), including the TA (Bacskai et al., 2014; McHanwell & Watson, 2009). In the present study, we did not attempt to directly evaluate TA MNs histologically, as the outcome using this type of preparation is far more variable compared to bona fide nerve dip approaches (Brandenburg et al., 2018). However, in aged Wistar rats, specific labelling of TA MNs has been achieved, with ~25% of MNs lost in old age (Ishihara et al., 1987). Motor unit number estimation approaches also show specific declines with age in human TA MNs (McNeil et al., 2005), although these are unable to distinguish denervation from MN death per se.

Our observations of lumbar MN loss in female and male rats, with no differences between sexes is consistent with prior lumbar MN observations in male F344 rats (Jacob, 1998). We also observe that lumbar MN loss with old age disproportionately affects larger (likely type FF motor units) MNs, consistent with observations in other MN populations in humans (Cruz‐Sanchez et al., 1998) and F344 rats (Cheung & Fogarty, 2026; Jacob, 1998). Larger MNs innervate type IIx/b fibers and comprise type FF motor units, while smaller MNs innervate type I and IIa fibers comprising type S and FR motor units, respectively (Burke et al., 1971; Fogarty & Sieck, 2019; Heckman & Enoka, 2012). The loss of larger lumbar MNs is consistent with the selective effects of aging on type IIx/b TA fibers. In other F344 MN populations, larger MNs have been shown to be vulnerable to death in old age (Fogarty, 2023; Fogarty et al., 2018; Fogarty et al., 2026; Fogarty & Sieck, 2023). In limb motor pools, the innervation ratio of type FF motor units is higher than those of type S and FR motor units (Rafuse et al., 1997). Thus, even small reductions in the number of type FF MNs may have outsized outcomes in terms of muscle fiber denervation.

One caveat to this interpretation is the lack of overall mean differences in lumbar MN size with age, which was not confounded by sex. Three reasons for this lack of overall reduction in surface area are potential contributors to this ambiguity. First, the transverse orientation of serial sections in the present study is required for adequate stereological evaluation and identification of the lumbar lateral motor column, but this plane is not consistent with the orientation of the largest extent of lumbar MN somas, that is, horizontal/parasagittal (Sterling & Kuypers, 1976). The second concern is the contribution of perinuclear lipofuscin to somal size, with MNs having increased abundance of lipofuscin with age, this may potentially cause some degree of somal swelling, regardless of MN type (Gray & Woulfe, 2005). Thirdly, surviving S or FR MNs may innervate type IIx/b muscle fibers denervated via MN loss, which may have retrograde consequences for MN identity. This would be consistent with the literature that suggests mixed fiber types and fiber type conversion occurs in limb motor units during aging and denervation (Carraro et al., 1985; di Maso et al., 2000; Rowan et al., 2012). Future studies are needed to confirm whether these size dependent differences exist when evaluating the largest somal extent of the lumbar MNs (Sterling & Kuypers, 1976), and whether these findings are consistent with a lack of proposed molecular identifiers of type FF MNs, (e.g., Chondroitin, metalloprotease 9) (Manuel & Zytnicki, 2019). Evaluation of MN population sizes within identified individual motor pools, similar to a suite of prior studies will also help to disambiguate this issue (Brandenburg et al., 2018; Fogarty et al., 2018; Fogarty & Sieck, 2023; Hashizume et al., 1988; Ishihara et al., 1987; Pannerec et al., 2016).

### 4.4 Links between muscle and MN activity and dysfunction in aging

**FIGURE 6:**
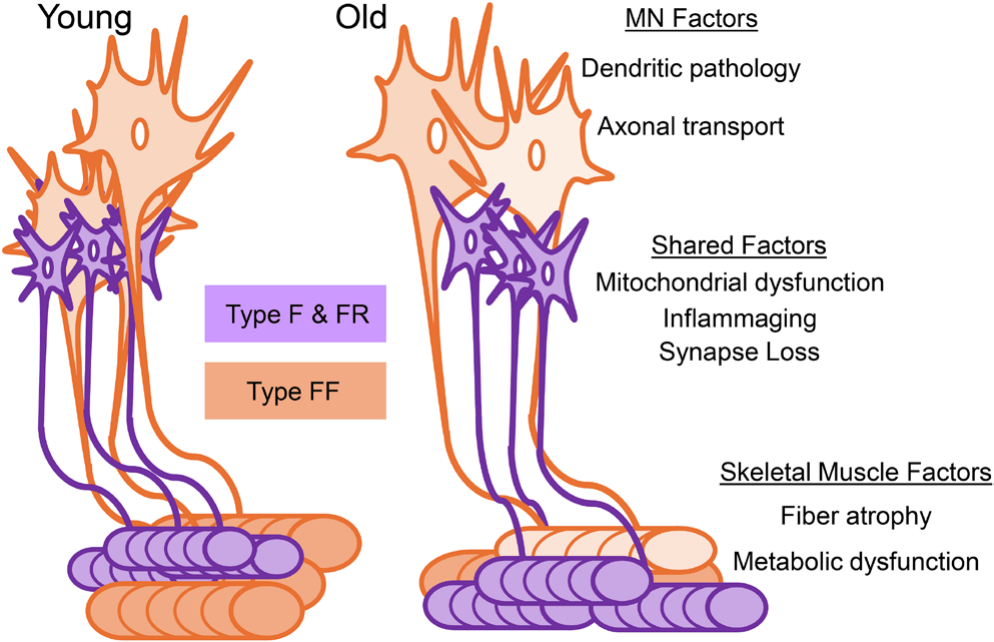
Proposed mechanisms underlying MN and muscle degeneration during aging. Age related MN degeneration is associated with dendritic pathology (Christensen & Fogarty, 2026; Coleman & Flood, 1987; Dickstein et al., 2013; Fogarty, 2025) and impaired axonal transport (Berth & Lloyd, 2023; Milde et al., 2015), which collectively contribute to MN vulnerability and death. Concurrently, muscle degeneration is characterized by progressive fiber atrophy (Larsson et al., 2019; Lexell et al., 1988; Nilwik et al., 2013) and metabolic dysfunction (Fattoretti et al., 2001; Fogarty, Marin Mathieu, et al., 2020; Grevendonk et al., 2021; Larsson et al., 2019; Proctor et al., 1995; Shou et al., 2020), resulting in reduced muscle mass and function. Several pathological mechanisms are shared between MNs and muscle, including mitochondrial dysfunction (Bua et al., 2006; Dupuis et al., 2009; Fogarty et al., 2026), central and peripheral (neuromuscular junction denervation) synapse loss and dysfunction (Castro et al., 2023; Christensen & Fogarty, 2026; Fogarty, 2025; Krishnan et al., 2018; Rowan et al., 2012; Valdez et al., 2012), and inflammaging (Castro et al., 2024; Fogarty et al., 2026; Katharesan et al., 2016; Piekarz et al., 2020; Sun et al., 2023). Together, these processes contribute to progressive MN degeneration and neuromuscular function observed in old age.

There are several shared pathophysiological pathways that occur in aging MNs and muscle (Figure 6). Prior to MN death and muscle sarcopenia, it seems that a variety of synaptic (Castro et al., 2023; Christensen & Fogarty, 2026; Coleman & Flood, 1987; Deschenes et al., 2010; Dickstein et al., 2013; Fogarty, 2025; Krishnan et al., 2018; Rowan et al., 2012; Valdez et al., 2012; Viteri et al., 2025), axonal transport (Berth & Lloyd, 2023; Milde et al., 2015), and mitochondrial disruptions (Bua et al., 2006; Dupuis et al., 2009; Fogarty et al., 2026) occur within the central nervous system, brainstem, and spinal cord MNs, and in the peripheral neuromuscular junctions and skeletal muscle fibers. Aside from these specific processes, a steady increase of inflammatory cytokines occurs in a generalized fashion (i.e., systemically), with accumulations of inflammatory cytokines occurring in neural and skeletal muscle tissues across the age‐span (Castro et al., 2024; Fogarty et al., 2026; Katharesan et al., 2016; Piekarz et al., 2020; Scheinert et al., 2015; Sun et al., 2023). These factors all influence muscle metabolism (Fattoretti et al., 2001; Fogarty, Marin Mathieu, et al., 2020; Grevendonk et al., 2021; Larsson et al., 2019; Proctor et al., 1995; Shou et al., 2020) and atrophy (Larsson et al., 2019; Lexell et al., 1988; Nilwik et al., 2013) in aging. The chronology and salience of each of these factors, and the degree to which they are cumulative or synergistic remains under intense investigation. This paper lends credence to the line that these pathophysiological mechanisms must occur in a framework that is consistent with motor unit‐specific degeneration (Figure 6).

A major push in the field has been to assess the effectiveness of a variety of lifestyle changes (caloric restriction and exercise) in preserving neuromotor function more generally and MN number and skeletal muscle strength more specifically. Caloric restriction and intermittent fasting have seized the imagination of many within and without the gerontological field. Despite the enthusiasm and seeming efficaciousness of caloric restriction as a means to maintain muscle health (Das et al., 2023; Weindruch, 1996), its use as a neuromotor panacea is not borne out under scrutiny, with unchanged MN survival and a gross decline in measures of MN health (many of which are shown to be improved in skeletal muscle) in calorie restricted compared to *ad libitum* fed groups (Chopek & Gardiner, 2010). Responses to exercise have created a degree of enthusiasm as a potential intervention for aging neuromotor decline. Despite well‐documented cardiovascular improvement and enhanced organismal fitness, neuromotor improvements stem from gains in the maintenance of gait and vestibular synaptic inputs to MNs (Battilana et al., 2020). Furthermore, whether exercise and activity is the salient factor, rather than a combination of social and environmental enrichment, is a major area of research (Prado Lima et al., 2018; Sampedro‐Piquero et al., 2018). More pertinent to MNs, exercise in ALS models has been shown to be contraindicated (Ma et al., 2017), despite the benefits of environmental enrichment (Sorrells et al., 2009). Clearly much work needs to be done to establish the necessary and sufficient factors derived from caloric restriction and exercise to have a robust, repeatable outcome, particularly in relatively sedentary models such as the laboratory rats used here.

### 4.5 Summary

In conclusion, the results of the present study illustrate age‐related morphological and metabolic vulnerability within type IIx/b TA muscle fibers. The alterations may underpin the selective vulnerability of type FF motor units. These findings were concomitant with disproportionate death of larger lumbar MNs (likely type FF). These findings support the premise that age‐associated neuromotor dysfunction and sarcopenia are motor unit type‐specific. This pattern of dysfunction and deficit observed in hindlimb muscle and lumbar MNs resembles those previously established in aging brainstem‐ (Fogarty, 2023; Fogarty et al., 2026; Sieck et al., 2023) and cervical‐innervated (Brown, Davis, et al., 2021; Fogarty et al., 2018; Fogarty, Marin Mathieu, et al., 2020; Gosselin et al., 1994) motor pools. Type FF motor unit vulnerability in normal aging is strikingly similar to age‐associated degenerative conditions afflicting MNs (e.g., Amyotrophic Lateral Sclerosis [ALS]) (Cheung & Fogarty, 2026). It has been tempting for us and others to speculate that common mitochondrial mechanisms trigger type‐specific neuromotor degeneration in both aging and ALS (Fogarty et al., 2018; Fogarty et al., 2026; Fogarty, Brown, & Sieck, 2020; Ham et al., 2026; Pandya & Patani, 2020; Valdez et al., 2012) (Figure 6). Our study suggests that strategies to maintain the oxidative capacity of vulnerable type IIx/b tibialis anterior fibers may help ameliorate age‐associated weakness and atrophy.

## AUTHOR CONTRIBUTIONS

**Kathryn D. Rooker:** Formal analysis; investigation; visualization. **Genesis A. Hernandez‐Vizcarrondo:** Data curation; investigation; visualization. **Sang Won Cheung:** Data curation; formal analysis; investigation; visualization. **Sanjana Mahadev Bhat:** Investigation; visualization. **Alyssa D. Brown:** Investigation. **Gary C. Sieck:** Funding acquisition; resources; supervision. **Matthew J. Fogarty:** Conceptualization; data curation; formal analysis; funding acquisition; investigation; project administration; supervision; visualization.

## FUNDING INFORMATION

This work was supported by National Institutes of Health Grants R01 AG044615 (GCS) and R01 AG086136 (MJF).

## CONFLICT OF INTEREST STATEMENT

None of the authors has any conflicts of interest, real nor perceived, to disclose. The funders had no role in the preparation of this manuscript.

## Data Availability

Data is available from the corresponding author at reasonable request.
